# Research advances on sake rice, koji, and sake yeast: A review

**DOI:** 10.1002/fsn3.1625

**Published:** 2020-05-19

**Authors:** Kaizheng Zhang, Wenchi Wu, Qin Yan

**Affiliations:** ^1^ College of Bioengineering Sichuan University of Science & Engineering Zigong China

**Keywords:** brewing, koji, sake rice, sake yeast

## Abstract

Sake is the national alcoholic beverage of Japan, and its history can be traced back more than 1300 years. With the development and maturity of the sake‐brewing technique, a unique flavor and taste gradually formed, which led to its wide use in Japan and internationally. This paper reviews and discusses the research advances of sake rice, koji, and sake yeast. The various enzymes and involved genes of microbes in the rice koji, and the separation/breeding of sake yeasts are expounded particularly. Moreover, the fields where further research is required are presented. Therefore, this review presents recent comprehensive research details of sake's ingredients and the involved study perspectives.

## Graphical abstract

Research progress of sake rice. Research progress of sake yeast and its breeding for these attributes: improved ethyl caproate and caproic acid productivity, nonurea production, ethanol‐resistant and nonfoam‐forming, etc.; Research progress of rice koji and it α‐amylase genes, etc. perspectives.

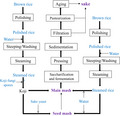

## 1 INTRODUCTION

Sake is a transparent moderate‐alcohol beverage of Japan, with a tradition lasting more than 1,300 years; it is brewed from nonsticky japonica rice grown in Japan and water by fermenting with the koji mold *Aspergillus oryzae* and sake yeast *Saccharomyces cerevisiae*. The alcohol content generally ranges from 13% to 17% vol. Sake possesses a clear color (Chinese rice wine is yellow or brown in color), a pure and pleasant taste, and an elegant flavor. It has an abundance of 18 amino acids that are beneficial to the human body and contains multivitamins, along with oligosaccharides, short peptides, and polyphenols. Moreover, the caloric content per 100 g of sake is as high as 109 kcal, which is 1.5 times more than that of wine and 2.5 times more than that of beer. Sake has positive healthcare effects, and studies reported that a specific amount intake of sake presented an anxiolytic effect (Izu, Yamada, Goto, & Sudo, 2010). In addition, sake concentrate and its specific sugar (α‐D‐glucoside ethyl ester) have been reported to inhibit chronic alcoholic liver injury (Izu, Hizume, Goto, & Hirotsune, 2008).

**Figure 1:**
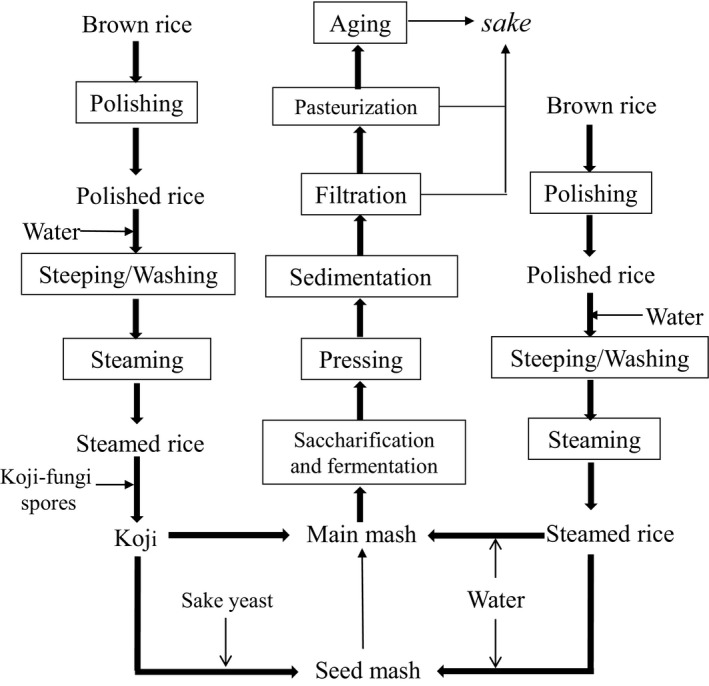
The brewing process for junmai sake

In Japan, more than 1,500 factories brew sake, among which the famous manufacturers included Kiku‐Masamune and Hakutsuru in Kobe, Gekkeikan in Kyoto, Nihonsakari and Ozeki in Nishinomiya. The raw material of Junmai sake includes sake rice, water, rice koji, and sake yeast, while the raw materials for synthetic sake are alcohol, rice, water, rice koji, sake yeast, sugar, flavor enhancer, and sour agent. After specific pretreatments, these raw materials (at reasonable ratios) were mixed with water to ferment for 2 weeks ~ 1 month at low temperature (below 15°C commonly), to obtain sake (Figure 1). Therefore, sake rice, koji, and sake yeast play a key role in the Japanese sake production.

## 2 SAKE RICE

Sake rice is the suitable rice type for sake making, and its quality is crucial for the quality of sake. Japanese rice varieties that are used for sake brewing are unique and show common characteristics such as large grains, low protein content, large white cores, high water absorption rate, high enzymatic digestibility of steamed rice, and low cracking ratio during polishing. The large white cores are classified into four types: white‐bellied core, ellipsoidal white core, lined white core, and dotted white core (Okuda, 2019). The expression of white core grains is not only governed by genetic factors of the rice cultivars, but also by environmental factors encountered during cultivation. Yamadanishiki cultivated in Japan's Hyogo Prefecture in 1923, was the most popular rice for sake making, and was widely planted. Its best producer was in Hyogo Prefecture, Japan. Yamadanishiki has a higher white core expression rate (WCE) and is lower in protein and fat compared with cooking rice cultivars. Okada et al. (2017) reported that the 100‐grain weight and WCE of Yamadanishiki were 1.30 and 7.74 times higher than those of Koshihikari (cooking rice cultivars), respectively. While the Yamadanishiki alleles at 16 QTLs contributed to larger grain size, two major QTLs that are essential for regulating both 100‐grain weight and grain width were harbored in the same regions on chromosomes 5 and 10. The QTLs associated with grain size also play an important role in the formation of white core, and the formation of white core does not depend on the grain‐filling speed (Okada et al., 2017). The Gohyakumangoku, which had large consumption among Japan's sakes, was surpassed by Yamadanishiki in 2001. Due to its large white core and frangibility, when it was ground by 50%, this rice was not suitable for daiginjo brewing. Gohyakumangoku rice had always been famous for its savory and elegant wine quality. Due to its strong cold tolerance, it was cultivated all over Japan. The Miyamanishiki rice was named since its white core looked like the white snow of the famous Alps in the Nagano Prefecture, and its production ranked third in Japan. Sake made from Miyamanishiki rice is fresh and has a pleasant fragrance (Yamamoto, 2017).

After harvest, the brown rice with large grain, white core, and without cracks is selected for grinding, and the resulting ground rice is called polished rice. The milled rice ratio refers to the proportion of polished rice in the original brown rice. Junmai sakes are generally divided into three grades according to the different milled rice ratio: The first grade is junmai daiginjo, which is brewed by pure rice with a milled rice ratio of 50% or less; the second is the junmai ginjo‐syu with a milled rice ratio of 60% or less; and the third grade is the junmai‐syu without the restriction of milled rice ratio. Since the protein content is higher in the outer layer than in the center of rice grains, and protein constitutes approximately 68% of the dry weight of brown rice grains, and 4%–6% of the dry weight of sake‐making rice grains with a 70% polishing ratio. If oligopeptides, amino acid, and unsaturated fatty acids are too high, negative effects, such as excessive coloration and/or unpleasant taste, may result. Hashizume et al. reported that five bitter‐tasting peptides, consisting of six to thirteen amino acid residues, were detected in sakes by RP‐HPLC and gel permeation chromatography (Hashizume, Okuda, Numata, & Iwashita, 2007). Peptide no. 17, whose C‐terminus was histidine, had the lowest threshold value (0.06 μM) and was described as having a particular “*zatsumi*” taste, and the C‐terminal residue of peptides for peptides nos. 13, 18, and 20 was determined to be proline, which has a hydrophobic side chain and conveys a negative taste with a bitter note since it is generally assumed that the bitterness of peptides is closely related to their hydrophobicity (Ney, 1971). Moreover, Hashizume et al. (2007) also reported that the ginjyo‐type sake made from highly polished (40%) rice grain had a lower level of bitter‐tasting peptides than the jyunmai‐type sake made from 70% polished rice grains (Hashizume et al., 2007).

During polishing, lipids, minerals, proteins, and moisture of white rice with 50% milling ratio can be reduced to about 4%, 18%, 55%, and 80% of the original amount, respectively. Brown rice contains 2.5%–3% crude fat and about 0.7% fat‐by‐hydrolysis obtained from phospholipids, proteolipids, and others. Because crude fat exists mainly in the germ and aleurone layer, milling greatly reduces the fat content below 0.1% in white rice while the fat‐by‐hydrolysis barely decreases (Yoshizawa, 1999). Therefore, the quality of the resulting sake is suggested based on the local statement in the Hyogo Prefecture as: “the whiter the rice, the better the sakes.”

Furthermore, Okuda et al. described that steamed sake rice grains, the starches which were low in amylose and had abundant short‐chain amylopectin showed high viscosity, little retrogradation, and low gelatinization temperatures. Furthermore, the enzyme digestibility of steamed rice grains in sake mash can be predicted by the mean air temperature during grain‐filling (one month after plant heading), and the prediction formula: *y* = −1.42x + 49.30 (*R* ^2^ = 0.748, *n* = 69), where y represents the enzyme digestibility of steamed rice grains, and x represents the mean air temperature during grain‐filling. These results can help to control sake production at an early stage of the sake‐making season (Okuda, Aramaki, Koseki, Inouchi, & Hashizume, 2006;Okuda, Aramaki, Koseki, Satoh, & Hashizume, 2005).

## 3 RICE KOJI

"The first step is making koji; the second is making yeast starter, and the third is brewing (mash)," remained the most refined summary of the sake‐brewing process of the Japanese for thousands of years, indicating that koji played a central role in the brewing of sake. 20% of rice from the raw material is soaked in water, steamed, inoculated with *Aspergillus*, and cultivated to obtain koji. Koji had three functions: One is to provide an enzyme source for the mash to dissolve and decompose the starch, protein, and fat in the rice; the second is to produce vitamins and amino acids, to offer nutrition for the sake yeast; and the third plays a important role in the flavor of sake (Akiyama & Zhou, 2002).

*Aspergillus oryzae* is the source of amylase and was adopted in Japanese koji making, which is a crucial fungus applied in traditional Japanese fermented food and beverage production. In 2005, its genome was sequenced (Machida et al., 2005). *A. oryzae* belonged to the same subspecies as *A. flavus*, but unlike *A. flavus*, *A. oryzae* does not produce aflatoxins. By studying the homologous cluster of aflatoxin biosynthesis pathway genes in *A. oryzae* RIB40, Tominaga et al. (2006) found that aflatoxin expression genes such as *avnA*, *verB*, *vbs*, and *omtA* in RIB40 were deleted, and thus, no aflatoxins were biosynthesized. In fact, long‐term application of *A. oryzae* in the food industry also proved its safety to some extent. The koji isolated from *A. oryzae* contained more than 50 types of enzymes, including proteases that can decompose proteins into amino acids, lipases that digest fat, and phytases that break down phytic acid and release a large amount of inorganic phosphorus. The most important enzyme is amylase. 1 g of pure amylase can saccharify 2 kg of starch within 1 hr at 50°C, indicating that amylase can saccharify 2000 times more starch than its own quantity. Under the synergy of various enzymes, *A. oryzae* can decompose the starch in rice into glucose, providing sufficient raw material for the subsequent fermentation process. To improve the yield and quality of sake, researchers have performed numerous studies about α‐amylase in *A. oryzae*. α‐amylase can act on both amylose and amylopectin by hydrolyzing the α‐1,4‐glycosidic bond inside the starch. The hydrolysates include dextrin, oligosaccharide, and monosaccharide, which can provide raw materials for the fermentation stage. The characteristics of α‐amylase were a decrease in viscosity of the solution and the disappearance of the iodine reaction. In 1894, the discovery of the peak amylase by Takamine laid the foundation for the entire amylase industry. In fact, *A. oryzae* strains usually possess the following three α‐amylase genes, namely, taka‐amylase genes: *amyA*, *amyB*, *and amyC*. The *amyB and amyC* genes are located on the chromosomes 5 and 3 of *A. oryzae*, respectively. They have the same nucleotide sequences, spanning approximately 5 kb, and including an incomplete transposon sequence at their 5´‐flanking region. The *amyA* gene, located on the chromosome 2, consists of 2.04 kb nucleotide sequences (with eight introns), which had one and two mismatches in the 5´‐flanking and coding regions, compared with *amyB and amyC* (Machida et al., 2005). In 1989, Tada et al. cloned the gene *amyA* of peak amylase (TAA) based on the genomic library of *A. oryzae*, which was then recombined into EcoR I. With the transformant containing the fragment, the activity TAA increased 2‐ to 5‐fold (Tada et al., 1989). In 1995, Morkeberg, Carlsen, & Nielsen (1995) determined intracellular and extracellular α‐amylase concentrations during the entire culture period of *A. oryzae*. High concentrations of glucose were found to inhibit the production of α‐amylase, while maltose could induce the production and secretion of α‐amylase. In 2000, the gene *amyR* was cloned and analyzed, which could explain the finding of Morkeberg. *amyR* codes a transcriptional activator involved in starch/maltose‐induced efficient expression of the amylolytic genes in *A. oryzae*. It encodes 604 amino acid residues, locating on the largest chromosome in *A. oryzae*, is about 1.5kb upstream of *agdA* (encoding α‐glucosidase) and transcribed in the opposite direction (Gomi et al., 2000).

Ito & Nessa exposed *A. oryzae* spores to γ‐ray mutagenesis and reported that the amylase production capacity of the mutant *A. oryzae* increased by 2‐ to 5‐fold, corresponding to the irradiation doses. In addition, the spore size and DNA content of the high‐yield mutant strain increased accordingly (Ito & Nessa, 1996). Soon afterward, Spohr et al. discovered that the α‐amylase production ability of *A. oryzae* to related to its morphological structure. Strains with dense mycelia presented a higher α‐amylase production capability (Spohr, Carlsen, Nielsen, & Villadsen, 1997). And then, Agger, Spohr, & Nielsen (2001) reported that the biomass concentration in the *A. oryzae* liquid culture correlated positively with the concentration of α‐amylase within a certain concentration range, while excessive concentration of biomass inhibited the production of α‐amylase due to a change of solution viscosity. Nemoto, Maruyama, and Kitamoto (2012) studied the contribution of three genes (*amyA*, *amyB*, and *amyC*) to the α‐amylase expression in *A. oryzae*. They reported that the contribution of *amyA* was lower than that of *amyB* and *amyC*. The reason for the large amount of amylase production by *A. oryzae* was the duplication of *amyB* and *amyC* genes.

The cell walls in plant cells are able to sequester the starch to prevent it from being hydrolyzed, while the enzymes that hydrolyze cellulose and hemicellulose in the cell wall are less expressed in *A. oryzae*. To improve the material utilization rate and reduce the slag in the brewing process, Sato et al. purified the hemicellulose‐degrading xylanase isoenzyme XynG2 from *A. oryzae* RIB218, which retains most activity after exposure to 80% alcohol for 30 min. When it is applied as an exogenous enzyme in the wine‐making process, the output of sake increased by 4.4% and the vinasse decreased by 4.6% (Sato, Fukuda, Zhou, & Mikami, 2010). In addition, the cellulase Cel‐2 isolated from solid medium by Yamane et al. can promote the decomposition of steamed rice and significantly improve the utilization of raw materials during the brewing process (Yamane, Fujita, Izuwa, et al., 2002; Yamane, Fujita, Shimizu, et al., 2002).

During the process of koji making, the reproduction of *Aspergillus* is affected by temperature and moisture. Hence, it is particularly significant to maintain constant temperature and moisture. Since the temperature within the rice is higher than its outside temperature, the reproduction of *Aspergillus* may be uneven. Prior to the mechanization era, the control of temperature and moisture mainly relied on manpower to mix rice koji, which required hard work. The wooden tool used for koji sometimes contained a substance called TCP (2,4,6‐trichlorophenol) originated from wooden tools treated with fungicides, which was a precursor of 2,4,6‐trichloroanisole (TCA), and the reason for the musty/muddy smell of sake (Miki, Isogai, Utsunomiya, & Iwata, 2005). Along with the advancement of technology, automatic koji machines were introduced instead of manpower to control the temperature of the product by supplying air with specific temperature and humidity. With the gradual increase in the curved box volume, the koji‐making efficiency was improved and the labor needed was decreased (Akao et al., 2011).

## 4 SAKE YEAST

Sake yeast refers to *S. cerevisiae* strains with different characteristics than other strains and with good suitability for sake brewing. Sake yeast can produce a higher concentration of ethanol than laboratory yeast during the brewing process, which is likely because the buoyant density and stress tolerance of the septic yeast cells in the stationary phase are lower than those in the laboratory yeast cells. After the cells stopped growing, it was difficult for sake yeast cells to enter a static state (Urbanczyk et al., 2011). Watanabe, Wu, et al. (2011)) studied this brewing characteristic at the genetic level and reported a deficiency of sake yeast in transcription factors Msn2 and/or Msn4. Furthermore, its environmental stress response is mediated by stress response element (STRE). The dysfunction caused by this deficiency increases the initial fermentation speed of sake yeast. Watanabe, Watanabe, Akao, and Shimoi (2009) constructed a *S. cerevisiae* strain with overexpression of Msn2 to obtain higher ethanol tolerance. Deletion of the *CLN3* gene significantly reduced the fermentation speed of *S. cerevisiae* and laboratory yeast. The *SWI6* gene, as a transcriptional activator, could promote the C1n3p‐mediated G1/S transformation in cells, the damage of which would decrease the fermentation rate. However, *S. cerevisiae* was a natural *whi* mutant that protected the above genes and played a significant role in the increase of the fermentation speed (Watanabe, Nogami, et al., 2011).

Japanese cultivates a variety of yeasts with different characteristics (Table 1) (Gu, 2016; Kitamoto, Oda‐Miyazaki, Gomi, & Kumagai, 1993; Kuribayashi, Sato, Joh, & Kaneoke, 2017). For instance, the K‐6 (Kyokai No. 6 yeast, similarly hereinafter) and K‐7 are officially distributed by the Brewing Society of Japan and produce their specific fragrance at low temperatures (10–12°C). K‐9 is suitable for the production of high‐grade sake, while K‐10 is suitable for brewing low‐acidity wine. K‐11 yeast has strong resistance to ethanol. K‐7 and K‐9 are the most widely applied strains in sake production. The K‐7 was isolated in 1946, showed good fermentation performance, and was commonly employed to make ordinary sake. K‐9 was isolated in 1953 and was often used to brew ginjo‐syu due to its fruity and refreshing taste. K‐601, K‐701, and K‐901 belong to nonfoaming yeasts where high foam does not form during the fermentation process. Hence, the space of the tank can be fully utilized, which improves productivity.

**Table 1:** Some isolated/breeded sake yeasts or the mutants (1900s‐2010s)

Strain ^a^	Sources/ Breeding method	Isolation/breeding time (Reference)	Strain phenotype
K1−5	Kiku‐Masamune, Gekkeikan, Sakura masamune brewery, etc./ Isolation	1906–1924 (Gu, 2016)	“Pure cultured yeast,” with improved fermentation property
K−6	Akita in the Tohoku area of Japan / Isolation	1935 (Gu, 2016)	Fragrance of ethyl caproate and isoamyl acetate, strong fermentation, and excellent brewing quality
K−7	Miyasaka brewery / Isolation	1946 (Gu, 2016)	Fragrance of ethyl caproate, strong fermentation, and excellent brewing quality
K−8	K−6 mutant/ Isolation	1970 (Gu, 2016)	More acid, Fragrance of ethyl caproate, higher fermentation temperature
K−9	Kumamoto prefecture/Isolation	1953 (Gu, 2016)	Low foam, Fragrance of ethyl caproate and isoamyl acetate
K−10	Fermented mash in the Tohoku area of Japan/Isolation	1951 (Gu, 2016)	Fragrance of ethyl caproate and isoamyl acetate; Less acid
K−11	K−7 mutant/ Isolation	1975 (Gu, 2016)	Slightly more acid; Less amino acids
K−12	Miyagi Prefecture/ Isolation	1965 (Gu, 2016)	Aromatic, fragrance of *ginjo* sake
K−13	K−9 and K−10/ Hybridization	1979 (Gu, 2016)	Aromatic, fragrance of *junmai* sake
K−601; K−701; K−901; K1001.	K−6, K−7, K−9, K−10/Mutagenizing	1969 (Gu, 2016)	Nonfoam‐forming; Less acid and good aroma
S−127; S−139	Shikoku sake brewery / Foam nonsticking	1969 (Gu, 2016)	Nonfoam‐forming; More acid
AA−60	K−11/ Mutagenizing	1977 (Gu, 2016)	Ethanol‐resistant and nonfoam‐forming
M9−4; M9−6; M10−4; M10−5.	K−9 and K−10 mutant/ Growth in a CAO medium^b^	1993 (Kitamoto et al., 1993)	Nonurea production
S9arg	Niigata Ginjyo no. 9/ Growth in a CAO medium and Cerulenin resistance	2017 (Kuribayashi et al., 2017)	Nonurea production and improved ethyl caproate productivity
C−8	G1103 mutant/ Mutagenizing by EMS and Leucine analog resistance	1991 (Ichikawa et al., 1991)	Improved ethyl caproate and caproic acid productivity
F−3; F−7.	*S. cerevisiae* RIB6002 and RIB6006/ Mutagenizing by EMS and Leucine analog resistance	1987 (Ashida et al., 1987)	Improved isoamyl acetate productivity
P33−17; P43−17.	K901/ Mutagenizing by EMS	2000 (Arikawa et al., 2000)	Improved isoamyl acetate productivity
K7‐VPA^LS^	K−7/ Growth in a valproic acid (VPA) containing medium	2017 (Tomimoto et al., 2017)	Improved isoamyl acetate productivity and lower isoamyl alcohol content
FAS2−1250S	K−7/ Self‐cloning	1999 (Akada et al., 1999)	Ethyl caproate‐overproducing
TDH3p‐ATF1	K−7/ Self‐cloning	2004 (Hirosawa et al., 2004)	Isoamylacetate‐overproducing
K−901‐mril‐SC‐D; K−901‐mril‐SC‐M.	K901/ Self‐cloning	2018 (Ikeda et al., 2018)	Low productivity of the DMTS‐P1 and DMTS ^c^

^a^ K1‐5: Kyokai no. 1–5; K‐6: Kyokai no. 6, and so on.

^b^ CAO medium: a medium contains canavanine, arginine, and ornithine.

^c^ DMTS‐P1: 1, 2‐dihydroxy‐5‐(methylsulfoxide) pentan‐3‐one; DMTS:. dimethyl trisulfide.

The Japanese sake industry attaches great importance to the breeding of sake yeast. Sake yeast breeding research was firstly conducted by Japanese researchers in combination with microbiology and genetic research methods in other fields. The isolation of yeast mutants had always been the main route for the selection of high‐quality yeasts. Ethyl caproate conveys a fruity aroma and is important for the quality of *ginjo* sake; however, the concentration of ethyl caproate is often insufficient during the brewing of *ginjo* sake. Therefore, a sake yeast that yields an increased amount of ethyl caproate became an intensive breeding target. Yeast cells synthesize ethyl caproate from ethanol and caproyl‐coenzyme A (CoA). Ichikawa et al. isolated a sake yeast mutant resistant to cerulenin, an inhibitor of fatty‐acid synthase. The isolated mutant (C‐8) produced about five times as much caproic acid and ethyl caproate as the control (G1103) during sake brewing (Ichikawa et al., 1991). Cerulenin‐resistant sake yeast carries a point mutation in the gene that encodes fatty‐acid synthase Fas2 and produces a considerable amount of caproic acid. Caproic acid or caproyl‐CoA are then conjugated with ethanol by the acyl‐CoA: ethanol *O*‐acyl transferases Eht1 and Eeb1 to form ethyl caproate. Today, this strategy to breed an ethyl caproate‐overproducing sake yeast is widely utilized to breed sake yeasts for the brewing of *ginjo* sake (Kitagaki & Kitamoto, 2013).

It is well known that sake fermentation with a nonfoaming yeast will decrease the production cost, because the volume of the required sake tank can be saved. Akiyama et al. isolated nonfoam‐forming yeast strains (S‐127, S‐139) from sake mashes and reported that nonfoam‐forming yeasts do not stick to foams though they had some weakness in attenuating power, high productivity of acid, and in the quality of production (Akiyama, Sugano, Kumagai, Saito, & Shimizu, 1969). Momose, Iwano, and Tonoike (1969) reported that the electrostatic binding of the cell walls of yeasts and lactic acid bacteria is responsible for the coflocculation of both yeasts and lactic acid bacteria. Shimoi et al. (2002) confirmed that the factor responsible for the foam formation of the mash by sake yeast is the cell wall protein Awa1, which is rich in serine and threonine residues, contains many repetitive sequences, and has the cell surface hydrophobicity to generate bubbles.

Ethyl carbamate (ECA) naturally occurs in fermented foods and beverages. It is spontaneously produced by the reaction between urea and ethanol, and is suspected to be a carcinogen at high doses in animal tests. Therefore, ECA levels in food products are to be reduced. Kitamoto et al. first succeeded in breeding a nonurea‐producing diploid sake yeast mutant by deleting the arginase gene *CAR1* on two chromosomes of sake yeast (K‐9), via genetic engineering. Then, they brewed sake without no urea with the gene *CAR1* deletion homozygous mutant, and no ECA was detected in the resulting sake, even after storage for 5 months at 30°C. The results indicated that ECA in sake originates mainly from urea, which is produced by arginase. Then, Kitamoto et al. (1993) used the *CAR1* mutant to develop a new medium for the positive selection of *CAR1* mutants, and many arginase‐deficient mutants could be easily isolated not only from a laboratory haploid strain, but also from sake yeasts and wine yeasts. No urea was detected in sake brewed with these isolated mutants, and no ECA formed during storage at 70°C for 10 hr. Most mutants had virtually the same fermentation profiles as their parent strain. The medium for the positive selection was as follows: Orn medium [0.17% yeast nitrogen base, 5 mM ornithine, 2% glucose] and Arg medium [0.17% yeast nitrogen base, 5 mM arginine, 2% glucose] were used for the confirmation of *CAR1* mutants. CAO medium [0.17% yeast nitrogen base, 10 mg/L canavanine, 5 mM ornithine, 1 mM arginine, 2% glucose] was used for the positive selection of *CAR1 *(Kitamoto, Oda, Gomi, & Takahashi, 1991;Kitamoto et al., 1993). The breeding of nonurea‐producing sake yeast was continuous until now. The gene *CAR1* encodes arginase and is located upstream of the FAS2 locus on chromosome XVI of *S. cerevisiae*. Kuribayashi et al. (2017) isolated nonurea‐producing sake yeasts from a FAS2‐G3748A (G1250S) mutant using a CAO‐selective medium after selection of FAS2 mutants with cerulenin. The resulting double mutants with strong ethyl caproate‐producing phenotype (As high as ~ 3.5 times that of the control) does not produce urea during sake fermentation, indicating it as a suitable candidate for the production of safe and high‐quality sake.

Isoamyl acetate has a banana‐like flavor and is one of the main ester flavors that determine the quality of *ginjo* sake. Therefore, the main focus was to improve the production of isoamyl acetate. Two major routes have been used for the biosynthesis of isoamyl alcohol. One is via α‐keto isocaproate in the L‐leucine synthesis pathway from glucose. The other is the so‐called Ehrlich mechanism from L‐leucine in rice and koji through transamination. In sake fermentation, the biosynthesis of isoamyl alcohol could either be inhibited or decreased by the accumulated L‐leucine. Ashida et al. reported a procedure to obtain the mutants of *S. cerevisiae* that produce sufficient isoamyl acetate, isolated from sake yeast RIB6002 and RIB6006. They used 5,5,5‐Trifluoro‐DL‐leucine as L‐leucine analog for the isolation to eliminate the feedback inhibition by accumulated L‐leucine. The concentration of isoamyl alcohol that was achieved with these mutants increased by about three or four times compared with the wild strain and reached about 666‐881ppm. A sufficient concentration of isoamyl acetate (25–29 ppm) was consequently obtained in the sake fermentation test of the mutants and wild strain (Ashida, Ichkawa, Suginami, & Imayasu, 1987). Arikawa, Yamada, Shimosaka, Okazaki, and Fukuzawa (2000) constructed diploid mutants from sake yeast by EMS treatment and selected three strains that produce a higher level of isoamyl acetate (15.5–17.8 mg/L), which were over twofold higher than that by the parent K901. In recent years, the screening of drug‐resistant mutants of sake yeast strains has been an effective method for the creation of superior strains. Tomimoto et al. reported that they succeeded to isolate a valproic acid (VPA)‐resistant mutant (K7‐VPA^LS^) of K‐7 using a VPA‐containing medium. The mutant was significantly more resistant to not only VPA‐induced cell death but also ethanol compared with the parent strain. Furthermore, the major characteristics of sake brewed by strain K7‐VPA^LS^ were lower amino acidity and higher isoamyl acetate content without increased isoamyl alcohol in comparison with/K7. Moreover, taste sensor analysis showed that the resulting sake has milder sourness and higher saltiness or richness than K‐7‐brewed sake. High isoamyl acetate production may be connected with a deficiency in phosphatidylinositol, which directly inhibits alcohol acetyltransferase, an enzyme responsible for isoamyl acetate synthesis (Tomimoto, Akao, & Fukuda, 2017).

The selection of *S. cerevisiae* by haploid hybridization was also commonly employed by researchers. Sake‐brewing yeast is a key microorganism in the production process of sake. The accumulation of alcohol in the fermentation process exerts toxic effects on yeast cells, thus inhibiting cell growth and metabolism as well as preventing the production of higher concentrations of alcohol. Kurose et al. treated haploid yeast/K701 with ethanol to screen for a haploid strain with resistance to 18% (vol) ethanol. A diploid strain was formed via hybridization, which could produce a large amount of fruity isoamyl acetate. This hybrid yeast was used in a sake‐brewing experiment at laboratory scale. A higher survival rate was achieved at the later stage of sake brewing, and the yields of isoamyl acetate and ethyl hexanoate were twice more than that of the parent strain (Kurose, Asano, Tarumi, & Kawakita, 2000). Yoshida et al. hybridized a low‐acid‐yield haploid strain of kyokai yeast with anticyanophycin haploidy, which produced high ethyl hexanoate and obtained *S. cerevisiae* strain Dp‐3–60 with high ester yield and low acid yield. Dp‐3–60 produced more ethyl hexanoate, less malic acid, and succinic acid than the commonly used K‐7 strain (Yoshida, 1995). Since cross‐breeding can simultaneously obtain excellent traits from both parents, the hybridization of haploid strains was a favorable breeding method to obtain diploid *S. cerevisiae*. In 1990, Tani et al. obtained diploid mater cells in the sake yeast/K9 by heat treatment coupled with random spore plating, and subsequently, polyploid cells (triploid, KK‐3; tetraploid, KK‐4) were constructed between the opposite mater cells by the mass mating technique and the isolation of the zygotes using a micromanipulator. The fermentation tests of the polyploid strains indicated that strains KK‐3 and KK‐4 could produce ethnal up to 18% vol (Tani, Tomohiro, Miyata, Furukawa, & Hayashida, 1990).

Protoplast fusion technology is an important strain improvement technology that originated in the 1960s. It refers to the process of parental cell fusion after removing the nucleus to obtain fused cells via exchange and recombination among genomes. The sake produced by *S. cerevisiae* YM39 contained lower amino acid and lower succinic acid contents than K‐701, while the malic acid and isoamyl alcohol contents were higher than in K‐701. Iwase et al. employed the protoplast fusion technology to fuse YM39 and K‐701 and isolated two stable tetraploid fusions. Wine‐making experiments showed that the sake brewed by fusion had lower amino acid acidity than K‐701, whose isoamyl acetate content was higher than that of the two parents (Iwase et al., 1995). Through protoplast fusion, yeast can also achieve better traits, such as high temperature resistance, improved maltose assimilation ability and pleasing taste of sake. Thus, it had good prospects in the wine industry.

"Hineka" refers to a bad smell of aged sake produced after storage. Dimethyl trisulfide (DMTS) is the main reason for "hineka," and its content is positively correlated with the total nitrogen concentration of sake. With high concentrations of sulfides, high concentrations of “hineka” may be produced, leading to odor after long‐term storage. Thus, inhibiting the production of DMTS is an effective way to reduce odor. Isogai et al. (2009), Isogai, Kanda, Hiraga, Iwata, and Sudo (2010) used cation exchange resin to separate the components in sake and reported that the precursor of DMTS was only present in acidic and neutral fractions. One of the precursor compounds 1,2‐dihydroxy‐5‐(methylsulfoxide)pentan‐3‐one (DMTS‐P1) was identified via ESI‐MS (Isogai et al., 2009, 2010). Quantitative analysis showed that the concentration of DMTS‐P1 in sake correlated positively with the concentration of DMTS during storage, indicating that DMTS‐P1 could promote the generation of DMTS (Okuda, Isogai, Joyo, Goto‐Yamamoto, & Mikami, 2009). Ikeda et al. established two strains (K‐901‐mril‐SC‐D, K‐901‐mril‐SC‐M) of sake yeast that lacked the MRI1 gene by a self‐cloning method, and the deletion of this gene greatly decreased the formation of DMTS‐P1. These strains were used in small‐scale sake‐brewing experiments, the results of which demonstrated that the concentrations of DMTS‐P1 in brewed sake were lower than the threshold value, while the concentrations of DMTS in the stored sake were greatly decreased. Furthermore, the sake components were almost identical as that of ordinary sake. Thus, the odor development during the sake storage was reduced and the quality of the resulting sake could be improved without affecting the properties of sake (Ikeda et al., 2018).

## 5 CONCLUSION AND PERSPECTIVES

Sake is the national alcohol beverage of Japan and is welcomed by consumers due to its refreshing taste and elegant flavor. The National Research Institute of Brewing (NRIB) of Japan has been conducting continuous research and innovations with regard to rice, yeast, *A. oryzae*, brewing water, production technology, and equipment for sake brewing. The flavor and texture of sake are also a perfect embodiment of the introverted, soft, and medium‐characteristic features of Japanese people. This paper reviewed Japanese sake rice, koji, and sake yeast. The characteristics of sake rice, various enzymes in rice koji, and the research advances of sake yeast were expounded in this review.

Various breeding methods and targets for sake yeasts exist, and the genetic engineering of sake yeasts has been attempted. Eliminating extraneous DNA sequences from the sake yeast genome represents the self‐cloning method, and the yeast strains generated with this method do not correspond to genetically modified organisms (GMOs). Therefore, this method will be a promising breeding strategy, which currently awaits public evaluation (Akada, 2002;Akada, Matsuo, Aritomi, & Nishizawa, 1999;Hirosawa et al., 2004;Iijima & Ogata, 2010;Kitagaki & Kitamoto, 2013). In fact, novel methods for the modification of the sake yeast genome without using genetic modification technology that achieves improved brewing performances should be developed.

The palatability of sake paired with different national or region foods could be an interesting and worthy topic for comprehensive exploration, which can further develop sake consumption in the world (https://www.nrib.go.jp/English/researches/research_topi.htm). Certainly, breeding and innovating sake rice cultivars, as well as the selection of novel sake yeast to produce higher‐quality sake or sake that meets the specific taste requirements of consumers in particular regions (countries), is a promising way to further promote sake in the world.

## CONFLICT OF INTEREST

The authors declare no conflict of interest.

## HUMAN AND ANIMAL RIGHTS

No animals or humans were used for studies that are the basis of this review.
